# Painful Bladder Syndrome/Interstitial Cystitis Successful Treatment with Montelukast: A Case Report and Literature Review

**DOI:** 10.7759/cureus.2876

**Published:** 2018-06-25

**Authors:** Muhammad Wajih Ullah, Sunita Lakhani, Sunder Sham, Afshan Rehman, Wardah Siddiq, Tariq Siddiqui

**Affiliations:** 1 Cardiology, Mayo Clinic, Rochester, USA; 2 Internal Medicine, Liaquat University of Medical and Health Sciences Hospital, Jamshoro, PAK; 3 Internal Medicine, Ghulam Muhammad Mahar Medical College, Sukkur, PAK; 4 Internal Medicine, Baylor Saint Luke's Medical Center, Houston, USA; 5 Internal Medicine, Beth Israel Deaconess Medical Center/Harvard Medical College, Boston, USA; 6 Internal Medicine, Maharashtra Institute of Medical Education & Research, Talegaon, IND

**Keywords:** bladder pain syndrome, montelukast, asthma

## Abstract

Painful bladder syndrome/interstitial cystitis (PBS/IC) is a chronic condition characterized by pelvic pain, urinary frequency, and urgency for more than six months in the absence of urinary tract infections. The etiology of PBS/IC is still an enigma. PBS/IC is challenging for doctors to diagnose because its symptoms overlap with other diseases such as urinary tract infection, overactive bladder, or endometriosis. Hence, it is diagnosed after excluding those diseases. The prognosis of PBS/IC may vary because of multiple treatment options. In this study, we are documenting a 26-year-old female patient who was successfully treated with montelukast after diagnosed with PBS/IC.

## Introduction

Painful bladder syndrome/interstitial cystitis (PBS/IC) is a disease of unknown etiology. The male-to-female ratio is 1:9 in the US population survey [1]. The International Continence Society (ICS) has defined PBS as "the complaint of suprapubic pain related to bladder filling, accompanied by other symptoms such as increased daytime and nighttime frequency, in the absence of proven urinary infection or other obvious pathology." The ICS uses the term interstitial cystitis for the symptom syndrome associated with typical cystoscopic and histological features [2]. PBS/IC is a diagnosis of exclusion and careful assessment of its symptoms, physical examination, urinalysis, urine culture, pelvic ultrasound, and cystoscopy with biopsy are indicated to differentiate PBS/IC from other causes of these symptoms.

Conservative treatments such as patient education, non-prescription medications, and pelvic floor exercises are considered for the management of PBS/IC. Although several oral treatment strategies for PBS/IC are being studied, their efficacy is questionable. The answer to effective treatment for PBS/IC needs to come from well-designed and accurate studies. In this study, we are reporting a patient presenting with PBS/IC successfully treated with montelukast.

## Case presentation

A 26-year-old patient presented to the clinic complaining of lower belly pain and increased urinary frequency for seven months. Her daytime urinary frequency ranged from 20 to 35 times with nocturia about six to eight times. The patient’s suprapubic pain was 6/10 in intensity and fluctuated quite markedly. She experienced no associated symptoms such as fever, weight/appetite changes, bowel habits, or any antecedent infection. The patient’s past medical history revealed she was a diagnosed asthmatic for the past 12 years and was on low dose inhaled corticosteroids. Her past surgical and family history was unremarkable, and she had no modifiable or non-modifiable risk factors. She had a mild allergic reaction to peanuts during her childhood. She never smoked cigarettes or used any illicit drugs. Obstetrics and gynecology history revealed she was gravida 0, her last menstrual period was two weeks ago, and she had no vaginal discharge. She was in a monogamous relationship with her boyfriend for four years and she experienced mild pelvic pain during sexual intercourse. Systemic examination was unexceptional except for suprapubic tenderness.

The patient was asked to follow-up with her urine and blood tests. Her urinalysis and urine culture were insignificant. Laboratory investigations including complete blood count, fasting blood glucose, Hemoglobin A1c, erythrocyte sedimentation rate (ESR), thyroid function tests, liver function tests were within normal limits. The patient was admitted to the hospital for additional investigations. Pelvic ultrasound and cystoscopy were insignificant. Common etiologies, which could lead to an increase in urinary frequency, urgency, or nocturia, are urinary tract infection, overactive bladder, and endometriosis which were excluded and the patient was diagnosed with PBS/IC. During her stay in the hospital, she experienced shortness of breath secondary to asthma exacerbation. The patient was managed by inhaled beta-2 agonists, anticholinergics, and oral corticosteroids. She was also given montelukast sodium (10 mg/d), and the patient reported a drastic improvement in her shortness of breath. The following day the patient noticed a decrease in her urinary frequency and urgency. She was discharged from the hospital on inhaled low dose corticosteroids and montelukast.

At her follow-up visit, two months later, the patient’s urinary symptoms were significantly reduced, and the suprapubic pain was gone.

## Discussion

**Figure 1:**
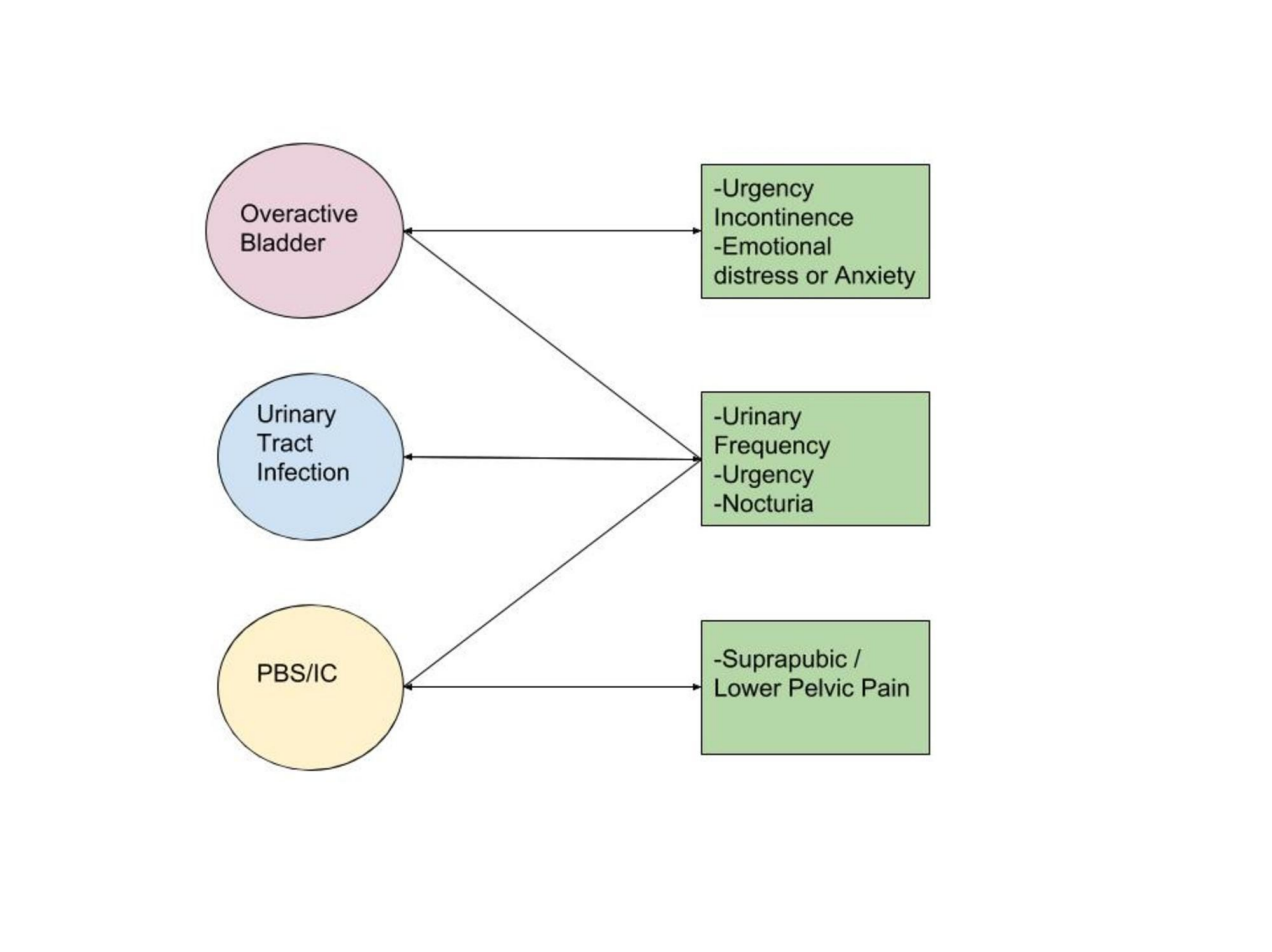
Differential diagnosis of Painful Bladder Syndrome/Interstitial Cystitis. PBS/IC: Painful Bladder Syndrome/Interstitial Cystitis.

PBS/IC is a term used by ICS to refer to a chronic symptom complex of the bladder characterized by lower pelvic pain, urinary frequency, and urgency for more than six months in the absence of urinary tract infections [3-4]. PBS/IC is more prevalent in females and is associated with various comorbidities such as allergies, fibromyalgia, endometriosis, and pain syndromes [5-9]. Therefore, a comprehensive history and a thorough physical examination are critical and should help to differentiate PBS/IC from other conditions that may present similarly, such as urinary tract infections, overactive bladder, endometriosis, and vaginal candidiasis (Figure *1*) [10].

Diagnosis of PBS/IC is based on the presence of classical urinary symptoms and suprapubic pain after excluding other alternative diagnoses. The investigations performed to reach a definite diagnosis are urinalysis, urine culture, pelvic ultrasound, and cystoscopy with biopsy. IC name is reserved for PBS with typical cystoscopic and histological features, whereas PBS includes pain in suprapubic or lower pelvic region. The National Institute of Diabetes and Digestive and Kidney Disease (NIDDK) established the findings of glomerulations or Hunner’s ulcers in the cystoscopy with hydrodistention as the diagnostic criteria for interstitial cystitis (IC) [11-12]. Positive biopsy findings of IC include inflammatory infiltrates and/or granulation tissue and/or detrusor mastocytosis and/or intrafascicular fibrosis [13].

The precise mechanism by which PBS/IC occurs is still unknown. However, it is believed that inflammation plays a central role in the pathogenesis of IC. One of the proposed mechanisms is the presence of leukotriene receptors in the detrusor muscle cells [14], increased urinary levels of leukotriene E4 in patients with PBS/IC and detrusor mastocytosis, suggesting a role of these proinflammatory mediators in IC [15]. Leukotriene E4 plays a role in the activation of mast cells and eosinophils. Once the mast cells are activated, they release vasoactive, inflammatory and nociceptive mediators, including kinin, leukotrienes, protease, prostaglandins, and nitric oxide [16-17], which are of utmost importance in chronic inflammatory disorders. Many symptoms and findings of IC such as pain, urinary frequency, edema, and fibrosis can be explained by mast cell-derived mediators. Montelukast, a cysteinyl leukotriene receptor-1 antagonist, has an anti-inflammatory role by inhibiting leukotriene receptors present in the bladder, thus, preventing the activation of mast cells.

**Figure 2:**
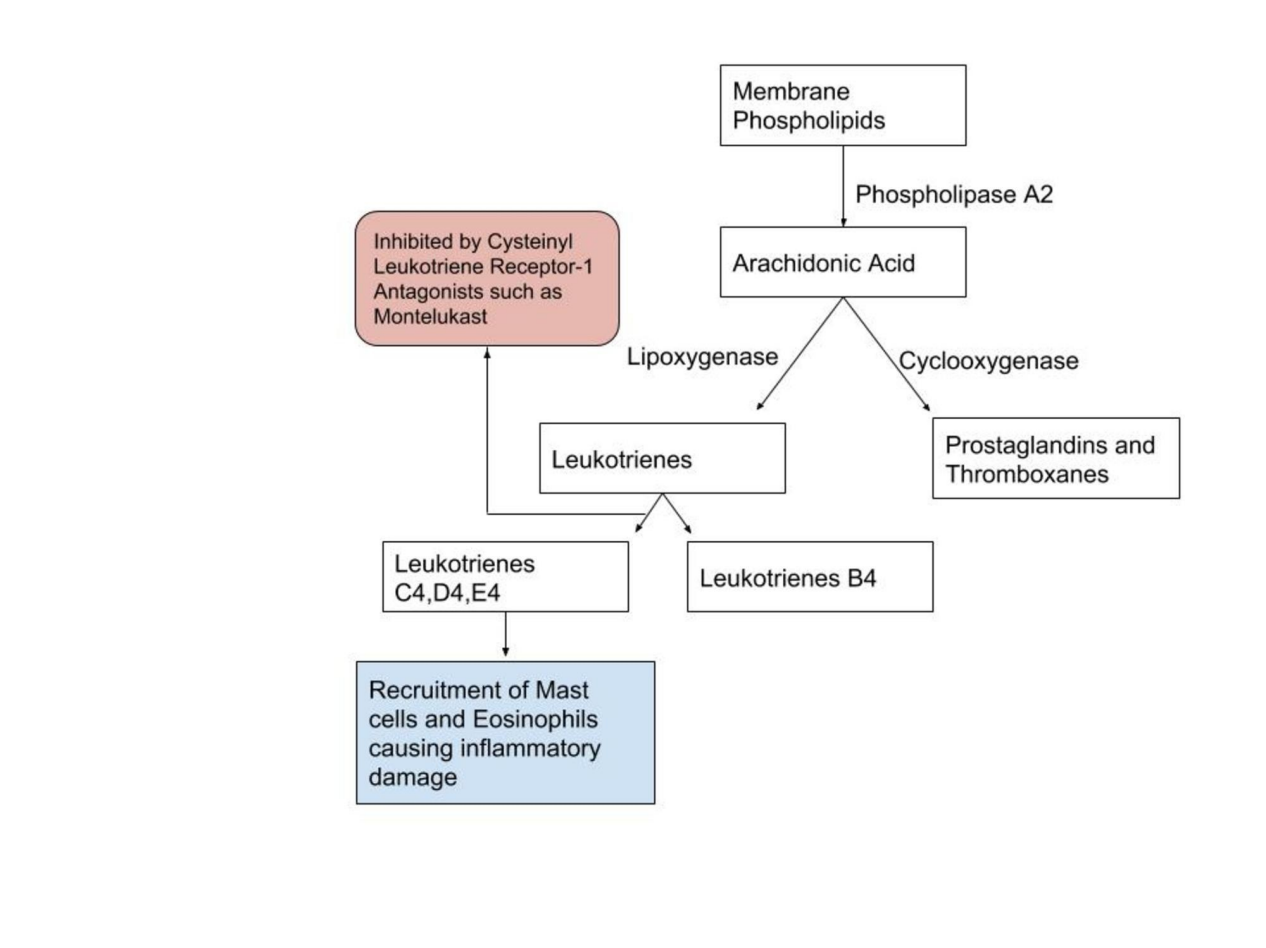
Leukotriene metabolites and their actions.

Our patient was diagnosed with PBS/IC and during her stay in the hospital, she had an exacerbation of her asthma. After stabilizing her, the patient was discharged with corticosteroids and montelukast. At her two months follow-up visit, the patient reported significant improvement in her urinary symptoms and suprapubic pain. We believe that montelukast is an effective treatment option for PBS/IC patients due to its anti-inflammatory effect by inhibiting recruitment of mast cells and eosinophils (Figure* 2*).

Similar results were observed by Bouchelouche et al. in a pilot study. Ten women with IC (diagnosed according to the NIDDK criteria and who also had detrusor mastocytosis with a minimum of 28 mast cells per mm) received 10 mg of montelukast daily for three months. After three months of montelukast treatment, there was a statistically significant decrease in the 24-hour urinary frequency, nocturia, and pain [18].

## Conclusions

PBS/IC is a not well-defined condition and its prevalence is increasing, especially among women. It is diagnosed clinically after excluding diseases which may present in an analogous way. The diagnosis is challenging and fewer effective treatments exist. In the context of this case study, the patient diagnosed with PBS/IC was successfully treated with montelukast, which may serve as a clue to support its role in PBS/IC treatment especially in patients with a history of allergic asthma.
